# Deregulation of SYCP2 predicts early stage human papillomavirus‐positive oropharyngeal carcinoma: A prospective whole transcriptome analysis

**DOI:** 10.1111/cas.12809

**Published:** 2015-10-16

**Authors:** Liam Masterson, Frederic Sorgeloos, David Winder, Matt Lechner, Alison Marker, Shalini Malhotra, Holger Sudhoff, Piyush Jani, Peter Goon, Jane Sterling

**Affiliations:** ^1^ Department of PathologyUniversity of CambridgeCambridgeUK; ^2^ Department of OtorhinolaryngologyCambridge University Hospitals National Health Service Foundation TrustCambridgeUK; ^3^ University College London Cancer InstituteLondonUK; ^4^ Department of HistopathologyCambridge University Hospitals National Health Service Foundation TrustCambridgeUK; ^5^ Department of Otolaryngology, Head and Neck SurgeryBielefeld Academic Teaching HospitalBielefeldGermany; ^6^ Department of DermatologyCambridge University Hospitals National Health Service Foundation TrustCambridgeUK

**Keywords:** Diagnosis by tumor markers and biomarkers, human papillomavirus, mRNA expression analysis, oropharyngeal carcinoma, Rb/p16‐related genes

## Abstract

This study was designed to identify significant differences in gene expression profiles of human papillomavirus (HPV)‐positive and HPV‐negative oropharyngeal squamous cell carcinomas (OPSCC) and to better understand the functional and biological effects of HPV infection in the premalignant pathway. Twenty‐four consecutive patients with locally advanced primary OPSCC were included in a prospective clinical trial. Fresh tissue samples (tumor *vs*. matched normal epithelium) were subjected to whole transcriptome analysis and the results validated on the same cohort with RT–quantitative real‐time PCR. In a separate retrospective cohort of 27 OPSCC patients, laser capture microdissection of formalin‐fixed, paraffin‐embedded tissue allowed RNA extraction from adjacent regions of normal epithelium, carcinoma *in situ* (premalignant) and invasive SCC tissue. The majority of patients showed evidence of high‐risk HPV16 positivity (80.4%). Predictable fold changes of RNA expression in HPV‐associated disease included multiple transcripts within the p53 oncogenic pathway (e.g. *CDKN2A*/*CCND1*). Other candidate transcripts found to have altered levels of expression in this study have not previously been established (*SFRP1, CRCT1, DLG2, SYCP2*, and *CRNN*). Of these, *SYCP2* showed the most consistent fold change from baseline in premalignant tissue; aberrant expression of this protein may contribute to genetic instability during HPV‐associated cancer development. If further corroborated, this data may contribute to the development of a non‐invasive screening tool. This study is registered with the UK Clinical Research Network (ref.: 11945).

Human papillomavirus (HPV) is strongly associated with the development of oropharyngeal carcinoma. Although over 150 genotypes have been described, HPV16 is considered responsible for ~95% of viral associated cancers at this site.^1^

Epidemiological evidence from the USA would suggest that HPV‐associated oropharyngeal squamous cell carcinomas (OPSCC) is rising at an ever‐increasing rate.^2^ If this published trend continues, the annual number of viral‐associated OPSCC cases will surpass cervical cancers by the year 2020. Global cancer statistics reflect this situation with a rise in incidence predominantly affecting younger adult males from developed nations.^3^

The roles of the two HPV16 oncoproteins E6 and E7 have been studied extensively and include, among others, inhibition of p53 and pRb (retinoblastoma) tumor suppressor proteins.^4^ This situation is quite different to HPV‐negative (HPV−) oropharyngeal SCC, where an irreversible *p53* mutation will normally be present and may contribute to the poorer clinical outcomes observed in this patient cohort.^5^, ^6^, ^7^ This information has provided the basis for several ongoing clinical trials that investigate de‐escalation treatment protocols in HPV‐positive (HPV+) disease.^8^, ^9^

Oropharyngeal squamous cell carcinogenesis involves progressive transformation of normal epithelium into premalignant tissue (dysplasia/carcinoma *in situ*) and, ultimately, invasive cancer.^10^, ^11^, ^12^, ^13^ Although the presence of HPV subtypes within invasive oropharyngeal SCC has been evaluated in large epidemiological studies,^1^, ^14^ there is limited data on this subject in regions of confirmed dysplasia/carcinoma *in situ*. Prior studies have reported markedly variable HPV prevalence rates due to limitations of size and variable assay techniques.^12^, ^15^ Jayaprakash *et al*. recently published a meta‐analysis of 22 relevant articles, suggesting HPV16 to be present in ~25% of all dysplastic lesions within the oropharyngeal subsite. The same author concluded this to be a conservative estimate due to the inclusion of oral cavity SCC lesions in some of the studies (traditionally a subsite with low HPV16 prevalence).^13^

The majority (~75%) of patients with HPV+ OPSCC present at an advanced stage (III/IV) due to cystic nodal disease.^7^, ^16^ In view of this, investigation of premalignant molecular pathways represents an important research priority, with the ultimate aim to produce a non‐invasive screening tool.^17^

## Materials and Methods

### Study population

This project received formal approval from the National Research Ethics Service Committee of East of England (12/EE/44). After informed consent, 24 consecutive patients with OPSCC donated multiple fresh biopsy samples (from regions of macroscopically normal and invasive tumor material) at Cambridge University Hospitals National Health Service Foundation Trust between June 2011 and July 2013. A further 27 OPSCC patients were included from a retrospective cohort and assessed for evidence of carcinoma *in situ* (dysplastic) change surrounding invasive carcinoma (Fig. S1). Disease stage was classified using the TNM classification of malignant tumors.^18^ Data from this study were deposited in the National Institutes of Health Gene Expression Omnibus database under accession code GSE56142. The trial protocol can be downloaded from the UK Clinical Research Network (http://public.ukcrn.org.uk).

In all prospective fresh biopsy samples, tumor and the adjacent normal tissue were processed for DNA and RNA extraction. A consultant histopathologist with expertise in head and neck pathology reviewed each sample to ensure representative sampling (minimum of 75% cancer cells for malignant tissue). Whole transcriptome analysis used the Illumina Genome Analyzer IIx machine (HumanHT‐12 version 4 BeadChip; Illumina, San Diego, CA, USA) and the results were validated with RT–quantitative real‐time PCR (RT‐qPCR) (ViiA 7; Applied Biosystems, Hampton, NH, USA).

The retrospective formalin‐fixed paraffin‐embedded (FFPE) tissue cohort was subjected to laser capture microdissection (LCM). This allowed precise RNA extraction from areas of invasive cancer, carcinoma *in situ*, and normal epithelial tissue to facilitate RT‐qPCR analysis.

### Human papillomavirus stratification

Human papillomavirus stratification methods included: consensus PGMY PCR, type‐specific HPV16 DNA PCR, and E6/E7 mRNA PCR, DNA sequencing, p16^INK4A^ immunohistochemistry (IHC), and HPV DNA *in situ* hybridization.

### Prospective cohort

Oropharyngeal fresh tissue samples from normal and invasive malignant regions (maximum 25 mg) were DNA extracted using a protocol published from this unit.^19^ As previously described, L1 DNA PCR analysis of tumor DNA (50–100 ng) involved the PGMY09/11 primer set with all negative samples subjected to further amplification using GP5+/GP6+ primers.^20^, ^21^ DNA bands identified after agarose gel electrophoresis were excised, purified using QiaQuick Gel Extraction columns (Qiagen, Venlo, Netherlands, UK) and sequenced directly (Source Bioscience, Cambridge, UK). The E6/E7 DNA and cDNA PCR analysis involved primers specific for HPV16 E6/E7.^22^ For all fresh tissue biopsies, parallel FFPE samples enabled p16^INK4a^ IHC (see below).

### Retrospective cohort

The p16^INK4a^ IHC was carried out on FFPE tissue using a mouse mAb (BD Biosciences, Franklin Lakes, NJ, USA).^23^ DNA *in situ* hybridization consisted of a probe directed against high‐risk HPV subtypes 16, 18, 31, 33, 35, 39, 45, 51, 52, 56, 58, and 66 (INFORM HPV III; Ventana, Tucson, AZ, USA).^24^ Genomic DNA was extracted from 9 × 3.5‐μm FFPE sections using a QIAamp tissue kit in accordance with manufacturer's guidelines (Qiagen). DNA was eluted in autoclaved and nuclease‐free H_2_O and stored at −20°C. Concentration and purity of DNA was assessed by spectrophotometry. Samples had an absorbance ratio (260/280 nm) in the range of 1.8–2.0, and were diluted with H_2_O to a concentration of 1–25 ng/μL prior to PCR. The L1 and E6/E7 DNA PCR was carried out as for fresh tissue samples (above).

**Figure 1:**
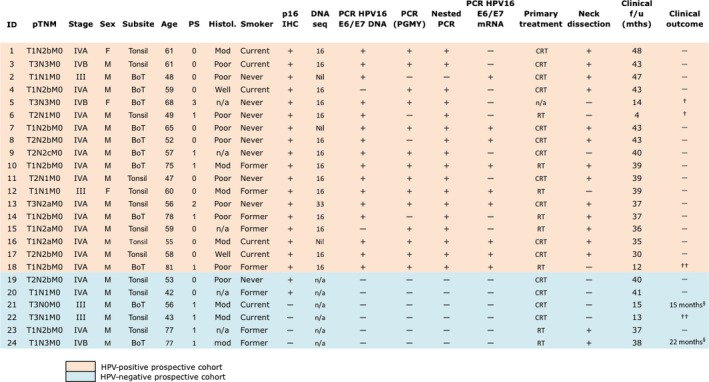
Clinical and histopathological data for the prospective cohort. Human papillomavirus (HPV)‐positive status was defined as evidence of HPV16 L1/E6/E7 DNA or HPV16 E6/E7 mRNA +/− p16^INK^ ^4A^ expression (>70% tumor cell staining). †Non‐malignant cause of death. ††Malignant cause of death. §Locoregional recurrence. BoT, base of tongue; CRT, chemoradiotherapy; F, female; f/u, follow‐up; Histol., histology; IHC, immunohistochemistry; M, male; Mod, moderately differentiated squamous cell carcinoma (SCC); mths, months; n/a, not applicable; Poor, poorly differentiated SCC; PS, Eastern Co‐Operative Group^(48)^ physiological performance status; RT, radiotherapy; Well, well differentiated SCC.

Clinical and histopathological data for all prospective and retrospective subjects are shown in Figures 1 and S1, respectively.

### RNA sequencing

The 24 OPSCC patients provided multiple fresh biopsy samples at the time of diagnostic or therapeutic surgery. All biopsies were selected on the basis of their RNA integrity number after histopathological review.

Messenger RNA‐seq cDNA libraries were prepared from ~400 ng total RNA. In brief, mRNA was isolated using polydT oligonucleotides connected to magnetic beads, fragmented using elevated temperature and divalent cations, and converted to cDNA using reverse transcriptase. DNA polymerase I and random primers were then used to convert single‐stranded cDNA into double‐stranded cDNA. This was blunt end repaired with Klenow DNA polymerase and T4 before adenylation of the 3′‐end of the fragment. A final purification step used gel electrophoresis, with fragments cut out in the range 200–300 bp. These fragments were amplified by PCR and sequenced using the Illumina Cluster Station and Genome Analyzer (Illumina). Paired‐end sequence analysis (51 cycles per end) was carried out with primers specific to the ends of the bridge‐amplified cDNA fragments to obtain 51 nucleotides of sequence from each end of all cDNA fragments.^25^

Each array on the HumanHT‐12 version 4 Expression BeadChip (GPL10558) targets more than 31 000 annotated genes with more than 47 323 oligonucleotide probes derived from the NCBI Reference Sequence Release 38 and other sources. Raw reads from normal epithelium (control) and tumor samples were processed using the GeneSifter Analysis Edition (Geospiza, Seattle, WA, USA) pipeline. Expression values for annotated genes were calculated from the aligned data by adding the number of reads linked to all exons and splicing events for a given gene and dividing that parameter value normalized by the total number of mapped reads in a sample. Two‐way anova identified target sequences with significant differential expression between normal and tumor tissue and further stratified by HPV status.^26^

### Reverse transcription–qPCR

Validation of expression data by RT‐qPCR analysis used the SYBR green method with an Applied Biosystems ViiA 7 Fast Real‐time PCR system. Primers (Eurofins MWG Operon, Ebersberg, Germany) were optimized with β*‐actin* as a control gene and then with the transcript region of interest. When the optimal primer concentration produced a linear response to input cDNA concentration (range, <1–150 ng), samples were analyzed in triplicate for each tested transcript.

### Statistical analysis

Statistical calculations were carried out using spss version 21 (SPSS, Chicago, IL, USA). To identify 80% of clinically relevant genes from the Illumina analysis, we based our power calculation on data supplied from Laborde *et al*.^25^ A minimal sample size of 10 subjects in each group was required if the false‐discovery rate (FDR) was set at 0.5% and the desired mean log2 fold change >1 (×10 change from baseline).^27^ Pearson's regression coefficient was used to investigate any correlation between the Illumina and RT‐qPCR analysis.^28^ Reverse transcription−qPCR data were analyzed by the 2^**−ΔΔ**CT^ technique, as described previously.^29^ In summary, the average C_t_ was derived for the three replicate analyses of the reference gene (β*‐actin*), and this was subtracted from the average C_t_ value from the three replicate analyses for the genes of interest. Expression differences between the HPV+ and HPV− tumors were compared using these normalized ΔC_t_ values and the observed differences subjected to a Student's *t*‐test. Rates of disease‐free survival (DFS) were estimated by means of the Kaplan–Meier method and were compared by the log–rank test. A multivariate model was developed using Cox regression to investigate the effect of clinical factors on disease outcome (HPV16, SYCP2, p16^INK4a^, SFRP1, T stage, N stage, sex, physiological performance status, oropharyngeal subsite, histology grade, smoking, concurrent chemotherapy, and age).

## Results

### Human papillomavirus stratification

In total, 18/24 of the fresh biopsy samples (prospective cohort) and 23/27 of the FFPE samples (retrospective cohort) were classified as HPV+, defined by evidence of HPV16 L1/E6/E7 DNA, HPV16 E6/E7 mRNA, or HPV DNA *in situ* hybridization episomal/integrative staining pattern. Immunohistochemical analysis for expression of p16^INK4a^ was shown for all HPV+ OPSCC samples but also present for 3 out of 10 OPSCC samples categorised as HPV− (Figs. 1,S1).

### Gene expression differences with HPV status in OPSCC

**Figure 2:**
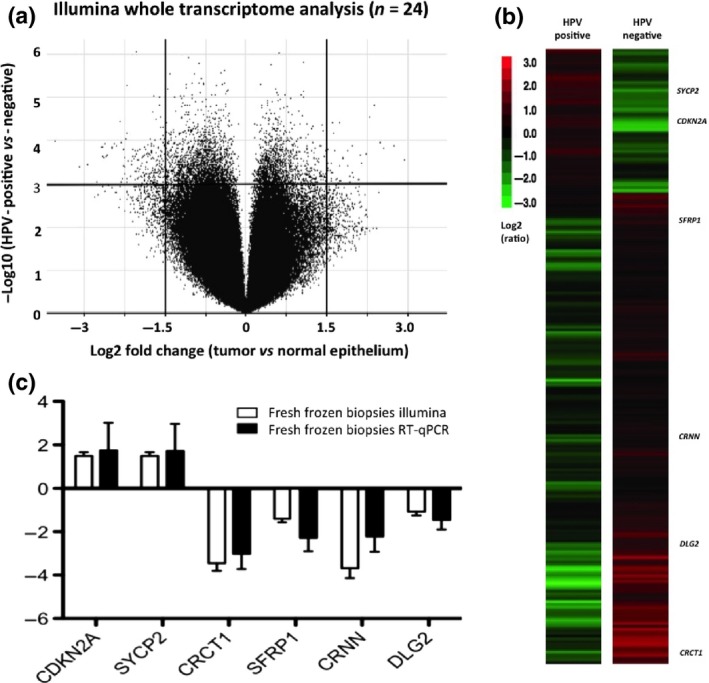
(a) Two‐way anova identified target sequences with significant differential expression between normal and tumor tissue and further stratified by human papillomavirus (HPV) status. In the volcano plot, the *x*‐axis is the log_2_ fold change value (tumor *vs*. normal epithelium) and the *y*‐axis is the −log10 odds value (HPV‐positive *vs*. HPV‐negative cohort). The two vertical lines represent 1.5 log2 (×15) fold change as the threshold cut‐off, both downregulated (left side) and upregulated (right side). The horizontal line represents a log odds value, *P* < 0.01 (false‐discovery rate of 0.5%), as the threshold cut‐off. (b) Hierarchical clustered heatmap of 223 genes displaying significant differential expression. As expected, *CDKN2A* ranked highly in the full transcriptome analysis. Five other genes that may have clinical relevance in HPV‐positive malignant disease are *SYCP2*,*SFRP1, CRCT1, CRNN*, and *DLG2*. (c) Validation graph for expression analysis (Illumina *vs*. RT–real‐time quantitative PCR [RT‐qPCR]; Pearson correlation coefficient, *r* = 0.905; *P* = 0.013; Kolmogorov–Smirnov test of normality, *P* > 0.10).

Among the 47 323 oligonucleotide probes on the DNA microarray, 223 differentially expressed genes were statistically significant in classifying HPV+ *versus* HPV− OPSCC (*P* < 0.01; FDR, 0.5%; Figs. 2a,b,S2). As expected, one of the most significantly expressed genes in HPV+ tumor tissue was *CDKN2A*, which encodes for p16^INK4A^. This cellular protein may be upregulated as a result of oncogenic HPV E7 inhibiting activity of pRb.^30^ Other genes noted to have significant differential expression in the HPV+ group and potential relevance in malignant disease are highlighted in Figure S3.

### Reverse transcription–qPCR

A subset of differentially expressed genes from the Illumina platform analysis was confirmed by RT‐qPCR (selected on the basis of clinical relevance in malignant disease). The six target transcripts were: *CDKN2A, SYCP2, SFRP1, DLG2, CRNN*,* and CRCT1*. A high level of agreement existed between the Illumina and RT‐qPCR analysis (Pearson's correlation coefficient, *r* = 0.905; *P*‐value = 0.013; Kolmogorov–Smirnov test of normality, *P* > 0.10) (Fig. 2c).

In the prospective fresh tissue cohort (Fig. S4), HPV+ OPSCC expression levels of *CDKN2A* and *SYCP2* were increased with an average log2 fold change of 1.7 (*P* < 0.01; 95% confidence interval [CI], 1.0–3.2) and 1.8 (*P* < 0.01; 95% CI, 1.35–3.35), respectively. Significant decreased expression was found for *CRCT1* (log2, −3.0; *P* < 0.001; 95% CI, 2.2–4.4], *SFRP1* (log2, −2.3; *P* < 0.01; 95% CI, 1.7–2.8), *CRNN* [log2, −2.2; *P* < 0.01; 95% CI, 1.7–2.9), and *DLG2* (log2, −1.5; *P* < 0.01; 95% CI, 1.4–2.2].

**Figure 3:**
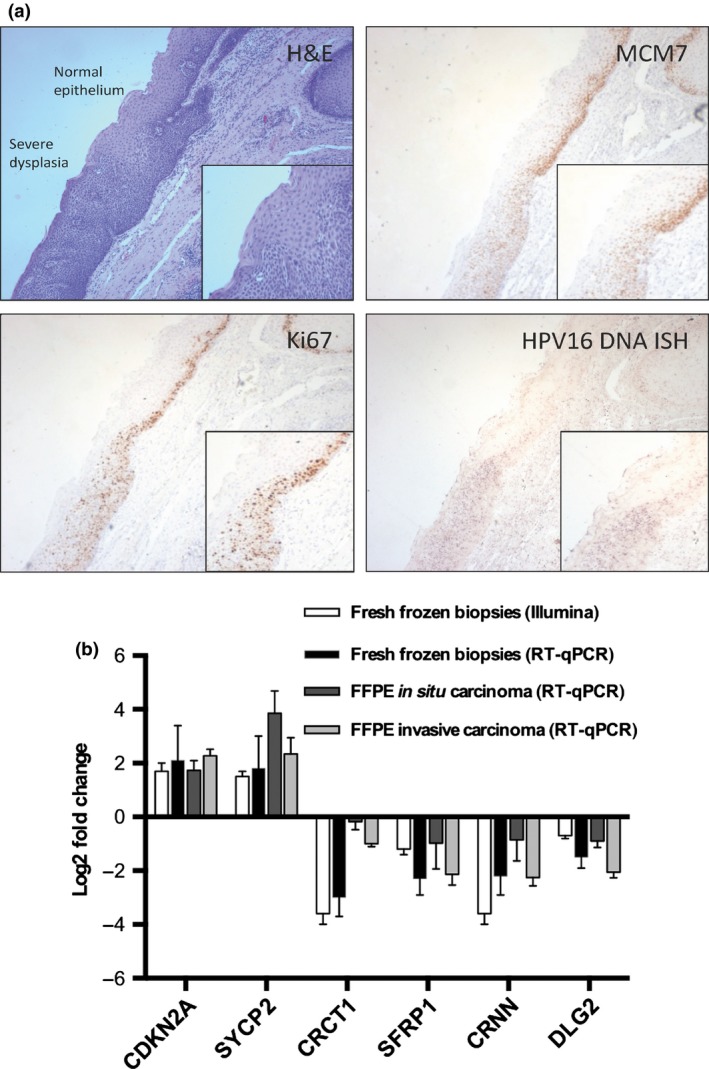
(a) H&E staining showing the junction between normal epithelium and severe dysplasia in human papillomavirus (HPV)‐positive tonsil squa‐mous cell carcinoma (top left). Although some flattening of cells at the apical surface may exist, the majority of the abnormal epithelium has a poorly differentiated dense basaloid appearance with nuclear pleomorphism. MCM7 (top right) and Ki67 (bottom left) staining are present in more than two‐thirds of the abnormal epithelium. *In situ* hybridization reveals an elevated level of HPV 16 DNA (bottom right) in the dysplastic epithelium when compared to the normal region (magnification, ×100; inset, ×150; Infinity capture software). ISH,* in situ* hybridization. (b) Reverse transcription–real‐time quantitative PCR (RT‐qPCR) analysis for all HPV‐positive sample types showing significant differential expression of *SYCP2* (log2 fold change, 3.1 [95% confidence interval, 1.8–4.4]; *P* < 0.01) in premalig‐nant (*in situ*) tissue.

Laser capture microdissection from the retrospective FFPE cohort allowed analysis of log2 fold change in adjacent regions of normal epithelium, invasive malignancy, and carcinoma *in situ* (Figs. 3a,S5,S6). The results largely reflect the analysis from fresh frozen samples for the HPV+ cohort; *SYCP2* (log2 fold change, 3.1; *P* < 0.01; 95% CI, 1.8–4.4) and *SFRP1* (log2 fold change, −0.97; 95% CI, −0.57–1.61) showed the largest differential expression from normal epithelium to premalignant (carcinoma *in situ)* tissue. In the same cohort, 70% of patients displayed significantly elevated expression of *CDKN2A* in the premalignant region (log2 fold change, >1.5). Figure 3(b) shows comparative data for both fresh and FFPE tissue designated within the HPV+ category.

### Clinical outcomes

**Figure 4:**
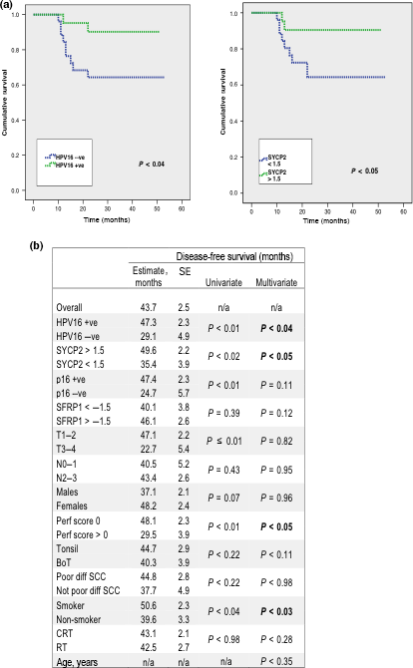
(a) Disease‐free survival calculations from Kaplan–Meier survival analysis of patients with human papillomavirus (HPV)‐positive oropharyngeal carcinoma. (b) A multivariate model was developed using Cox regression to investigate the effect of clinical parameters on disease‐free survival. Strati‐fication by HPV16, SYCP2, physiological perfor‐mance status, and smoking were all retained in the final model [bold text p < 0.05]. +ve, positive; −ve, negative; BoT, base of tongue; CRT, chemoradiotherapy; n/a, not applicable; SCC, squamous cell carcinoma; (SCC); mths, months; Perf, physiological performance status; Poor diff, poorly differentiated SCC; RT, radiotherapy.

The average DFS for all patients was 43.7 months (+/− SE, 2.5). For the HPV16+ cohort, the DFS period increased to 47.3 months (+/− SE, 2.3). Significant expression of SYCP2+ (log2 fold change, >1.5) and SFRP1+ (log2 fold change, <1.5) cohorts showed an average DFS period of 49.6 months (+/− SE, 2.2) and 40.1 months (+/− SE, 3.8), respectively. A multivariate proportional hazards model using Cox regression analysis revealed HPV16, SYCP2, smoking, and physiological performance status to have significant influence on DFS (Figs. 4,S7).

To evaluate the prognostic accuracy of *SYCP2* expression in regions of HPV+ *in situ* carcinoma, we constructed a receiver operating characteristic (ROC) curve (Figs. S8,S9). The area under the ROC curve was found to be 0.86 (+/− SE, 0.08; 95% CI, 0.71–0.99), indicating a good discriminating power when compared to control subjects. Sensitivity and specificity estimates over a range of cut‐off points suggest optimal results were obtained for the log2 fold range 1.5–3.0 (sensitivity, 70%; specificity, 95%). Similar testing of *SFRP1* expression revealed the area under the ROC curve to be 0.64 (+/− SE, 0.11; 95% CI, 0.42–0.86), indicating a poor discriminating power when compared to controls.

## Discussion

In this prospective observational study, we describe the use of mRNA massive parallel sequencing technology to investigate HPV+/− OPSCC tumors *versus* matched normal tissue. The data obtained were then validated by RT‐qPCR and the results used on retrospective FFPE tissue to investigate premalignant change surrounding areas of invasive carcinoma.

To our knowledge, comparatively few studies have investigated expression profiles in HPV‐associated OPSCC,^29^, ^31^, ^32^, ^33^ and even fewer have focused on precancer pathways.^34^ In particular, further characterization of a pre‐malignant state in the development of HPV‐associated OPSCC would be of clinical value as it infers the potential for a screening test (similar to the cervical carcinoma model). In HNSCC surgical excisions, dysplastic epithelium is often found adjacent to the cancer,^10^ but the exact nature of the disease in these regions is poorly defined.^12^, ^13^

Within our retrospective LCM cohort, 70% of patients showed significantly increased expression of *CDKN2A* (a proxy marker for HPV infection) in regions of carcinoma *in situ* relative to normal epithelium. This is higher than the HPV16 estimate (~25%) provided by Jayaprakash *et al*.^13^ and may be consistent with the hypothesis that HPV plays a significant role in the early phase of oropharyngeal carcinogenesis. Of course, high‐risk HPV infection can be a transient phenomenon and detection alone may not be sufficient to provide a causal association. Many studies have previously shown the presence of HPV subtypes even in normal oral cavity tissue.^35^, ^36^, ^37^ Gillison *et al*. (2012) carried out the largest epidemiological study on this topic (~6000 subjects), and estimated the prevalence for high‐risk HPV subtypes to be 6.9%, of which ~1% can be attributed to HPV16.^38^

It should still be noted that high‐risk HPV subtypes in normal oral cavity samples have significantly lower prevalence than the estimates reported by Jayaprakesh *et al*. (and largely confirmed in this present study). Available published reports also suggest a higher prevalence of HPV16 within areas of *in situ* malignant change when stratified by the male sex (×2 compared to females) and between areas of transformation from normal through to dysplastic epithelium (×3).^13^

In our prospective cohort of 24 OPSCC patients, RNA massive parallel sequencing data provided a statistically significant association for 223 target transcripts stratified by tumor and HPV status. Gene ontology data revealed the majority of transcripts to have limited clinical relevance but a focused analysis of oncological pathways produced a number of possible transcript candidates e.g. *SYCP2, SFRP1, DLG2, CRNN*, and *CRCT1*. Of these, *SYCP2* showed the most significant change from baseline in premalignant retrospective FFPE tissue, superseding the performance of *CDKN2A* (encoding for p16^INK2A^).

**Figure 5:**
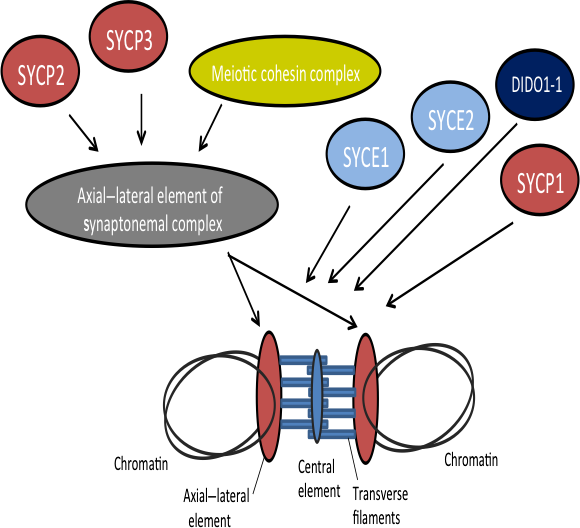
Hypothetical model for SYCP2 with regard to human papillomavirus‐positive oropharyngeal squamous cell carcinomas. SYCP2 is a protein that is involved in linkage chromosomes through the synaptonemal complex, binding DNA at the scaffold attachment regions and driving the prophase of meiosis. Alterations in the gene expression of *SYCP2* have been associated with impaired meiosis.

The elevated expression of *SYCP2* in HPV‐associated tumor tissue has previously been noted in three expression analysis studies.^29^, ^32^, ^39^ *SYCP2* is a testis‐specific human gene and aberrant expression in HPV+ cancers may contribute to the genomic instability induced by high‐risk HPVs and subsequent oncogenic change.^31^ A hypothetical model applied to HPV+ oropharyngeal carcinoma is provided in Figure 5. The Wisconsin Alumni Group Foundation has included *SYCP2* as one of three target biomarkers in the development for OPSCC.^40^ To date, no other study has revealed its elevated expression in premalignant tissue. The use of *SYCP2* as a prognostic indicator is also of interest, given our DFS data, however, further research will be required to discern if this is truly independent of HPV16 expression.

The p16^INK4a^ protein is an inhibitor of cyclin‐dependent kinase and has increased expression with elevated levels of HPV E7, however, many units have reported a concern regarding false positive results.^41^ Within our HPV16 negative cohort (10/51), three patients had elevated expression of p16^INK4a^, which may concur with this analysis. At present, stratification of OPSCC tumors by p16^INK4a^ alone is still the preferred approach by the majority of oncology centers. In an era of OPSCC de‐escalation treatments, which are based on a viral etiology, this may incur a risk of undertreating a small proportion of patients falsely considered as HPV+.^(48)^ The use of stepwise algorithms, which combine different HPV assays, may compensate for the limitations of individual tests and should now be considered in clinical settings.^42^

In this study, positive smoking status proved to have a significant adverse effect on DFS, regardless of HPV category (multivariable analysis, *P* < 0.03). The negative impact of smoking in HPV+ OPSCC has previously been highlighted by several randomized trials with *post‐hoc* analysis of HPV status.^6^, ^43^, ^44^ All studies indicated that the degree of tobacco exposure at diagnosis and during treatment directly correlates with the risk of disease progression and death from malignancy. This may indicate that smoking confers additional tumor mutations in the HPV+ cohort, leading to more aggressive disease and inferior responses to available curative‐intent therapies.

However, important questions remain about how to quantify smoking risk to enable comparison between studies. All the major de‐escalation trials have largely adopted the arbitrary cut‐off point proposed by O'Sullivan *et al*.^45^ in which ‘smokers’ are defined as having >10 pack year history. Perhaps more reliable information can be obtained from Laborde *et al*.,^25^ who recently published transcription profile data in OPSCC patients stratified by both HPV and smoking status. This indicated that two genes involved in the p53 DNA damage repair pathway, *ATR* and *CHEK2*, display patterns of increased expression associated with HPV− OPSCC smokers only.

This study is limited by its exclusive focus on whole transcriptome analysis; we recognize the need to integrate DNA sequence analysis in future projects.^11^ DNA analysis will indicate changes that have occurred to the DNA sequence, whereas mRNA sequence analysis clarifies the effect of those changes. This critically important process therefore identifies which mutations and rearrangements could be the best diagnostic and prognostic indicators. Of course, many current studies display the importance of DNA sequencing in establishing mutations associated with cancer development. The final choice of six target transcripts in this study may also be open to debate as it is primarily based on gene ontology data supplied through the KEGG^46^ pathway network.

In conclusion, developments in whole‐genome sequencing and mRNA analysis are rapidly creating an opportunity to provide personalized information on genetic and functional aspects of malignant tumors. With regard to HPV+ OPSCC, the investigation of differentially expressed genes in normal, premalignant, and malignant tissue may reveal unique pathways that can explain their different natural history and biological properties. The data from this study reveal *SYCP2* as a potentially significant biomarker; if corroborated on a larger scale this may contribute to the development of a non‐invasive screening tool, e.g. mouthwash or brush biopsy. Current epidemiological data would suggest it is not sufficient to simply screen for OPSCC by the presence of HPV16 alone (due to a ~1% carriage rate in the general adult population). Clearly, further well‐designed prospective studies are required to confirm this data and also to establish if other biomarkers may have future significance.

## Disclosure Statement

The authors have no conflict of interest.

## Supporting information


**Fig. S1.** Clinical and histopathological data for retrospective cohort of oropharyngeal squamous cell carcinoma patients. Human papillomavirus (HPV)‐positive status was defined as evidence of HPV16 L1/E6/E7 DNA or episomal/integrative pattern on HPV DNA *in situ* hybridization +/− p16^INK4A^ expression (>70% tumor cell staining). †Non‐malignant cause of death. ††Malignant cause of death. #Locoregional recurrence. BoT, base of tongue; CRT, chemoradiotherapy; F, female; f/u, follow‐up; IHC, immunohistochemistry; ISH, *in situ* hybridization; M, male; Mod, moderately differentiated SCC; mths, months; n/a, not applicable; pal., palate; Poor, poorly differentiated SCC; RT, radiotherapy; SCC, squamous cell carcinoma; Well, well differentiated SCC.

**Fig. S2.** Full heatmap of 223 transcripts that are differentially expressed between human papillomavirus (HPV)‐positive and HPV‐negative oropharyngeal squamous cell carcinoma tumors (*P* < 0.01; false‐discovery rate 0.5%).

**Fig. S3.** Gene ontology data for the 6/223 transcripts with possible oncological relevance.

**Fig. S4.** Reverse transcription–real‐time quantitative PCR data for prospective human papillomavirus (HPV)16‐positive and HPV16‐negative cohorts (fresh tissue). When the optimal primer concentration produced a linear response to input cDNA, samples were analyzed in triplicate for each tested transcript (*CDKN2A*,* CRCT1*,* SYCP2*,* SFRP1*,* CRNN*, and *DLG2*). β‐Actin (*ACTB*) was used as the housekeeping gene.

**Fig. S5.** Reverse transcription–real‐time quantitative PCR data for retrospective cohort (laser capture microdissection invasive *vs*. normal tissue). Formalin‐fixed paraffin‐embedded tissue samples were subjected to laser capture microdissection to enable RNA extraction from representative regions of invasive carcinoma and adjacent normal tissue.

**Fig. S6.** Reverse transcription–real‐time quantitative PCR data for retrospective cohort (laser capture microdissection carcinoma *in situ vs*. normal tissue). Formalin‐fixed paraffin‐embedded tissue samples were subjected to laser capture microdissection to enable RNA extraction from representative regions of *in situ* carcinoma (premalignant change) and adjacent normal tissue.

**Fig. S7.** Disease‐free survival – multivariable and univariable statistical analysis. A proportional hazards model using Cox regression analysis revealed human papillomavirus (HPV)16, SYCP2, smoking, and physiological performance status to have significant influence on disease‐free survival.

**Fig. S8.** Receiver operating characteristic (ROC) curve to evaluate prognostic accuracy of SYCP2/SFRP1 biomarker expression in regions of human papillomavirus‐positive (HPV+) *in situ* carcinoma. All control subjects were patients undergoing tonsillectomy for benign pathology (see Fig. S9). A further analysis incorporated data from the HPV− oropharyngeal squamous cell carcinomas cohort.

**Fig. S9.** Reverse transcription–real‐time quantitative PCR data for the prospective control cohort. Fresh tonsil biopsy samples were subjected to RNA extraction (epithelium and adjacent stroma region).
