# Supplementary figures and images for: Deregulation of SYCP2 predicts early stage human papillomavirus‐positive oropharyngeal carcinoma: A prospective whole transcriptome analysis
Source: Cancer Sci. 2015 Oct 16;106(11):1568–75. doi: 10.1111/cas.12809 (PMC4714680; doi:10.1111/cas.12809)

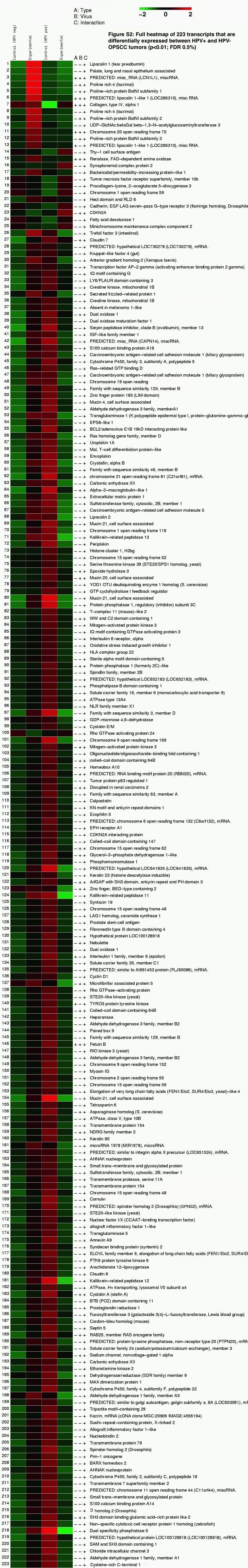

Supplement: Supplementary file 2 — Fig. S2. Full heatmap of 223 transcripts that are differentially expressed between human papillomavirus (HPV)‐positive and HPV‐negative oropharyngeal squamous cell carcinoma tumors (P < 0.01; false‐discovery rate 0.5%). [file CAS-106-00000000001568-s002.jpg]
