# Supplementary material for: Deregulation of SYCP2 predicts early stage human papillomavirus‐positive oropharyngeal carcinoma: A prospective whole transcriptome analysis
Source: Cancer Sci. 2015 Oct 16;106(11):1568–75. doi: 10.1111/cas.12809 (PMC4714680; doi:10.1111/cas.12809)
Supplement: Supplementary file 3 — Fig. S3. Gene ontology data for the 6/223 transcripts with possible oncological relevance. [file CAS-106-00000000001568-s003.docx]

**Figure S3:** Gene ontology data for the 6/223 transcripts with possible oncological relevance

| **Gene symbol** | **Rank (1-311)** | **Description (novel transcript in HPV+ OPSCC)** |
| --- | --- | --- |
| **CRNN** | **169** | CRNN [cornulin) is a recently identified gene encoding a protein with an S100 EF-hand calcium-binding motif, and its expression is known to be down-regulated in esophageal squamous cell carcinoma. CRNN overexpression in oral squamous carcinoma negatively regulates cell proliferation by the induction of G1 arrest. |
| **CRCT1** | **223** | CRCT1 (cysteine-rich C-terminal 1) is a protein-coding gene, and has been validated by a similar gene expression profiling study for cervical carcinoma. Down regulation of CRCT1 [also known as NICE1] may predispose to oncogenic change. |
| **SFRP1** | **35** | This gene encodes a member of the Secreted Frizzled Related Protein (SFRP) family that contains a cysteine-rich domain homologous to the putative Wnt-binding site of Frizzled proteins. Members of this family act as soluble modulators of Wnt signaling; epigenetic silencing of SFRP genes leads to deregulated activation of the Wnt-pathway, which is associated with cancer. The role of SFRP1 as a tumour suppressor has been proposed in many cancers based on its loss in patient tumors. |
| **DLG2** | **218** | This PDZ domain gene has not been investigated previously but DLG1 and DLG4 have demonstrated a significant interaction with HPV16 E6 in cervical carcinoma. This gene encodes a member of the membrane-associated guanylate kinase (MAGUK) family. The encoded protein forms a heterodimer with a related family member that may interact at postsynaptic sites to form a multimeric scaffold for the clustering of receptors, ion channels, and associated signaling proteins. |
| **SYCP2** | **16** | SYCP2 (synaptonemal complex protein 2) is a protein-coding gene. SYCP2, an SYCP1 homologue, is a component of the meiotic synaptonemal complex like structure. Increased expression of these meisosis specific proteins in testicular cancer may contribute to the genomic instability and subsequent oncogenic change. |
| **Gene symbol** | **Rank (1-311)** | **Description (established transcript in HPV+ OPSCC)** |
| **CDKN2A** | **32** | Cyclin-dependent kinase inhibitor 2A (CDKN2A) is also known as P16^INK4A^. It is a tumor suppressor protein that is encoded by the CDKN2A gene. CDKN2A / P16^INK4A^ plays an important role in regulating the cell cycle and is significantly over-expressed in response to HPV E7. |
| **CCND1** | **136** | The CCND1 gene encodes for the protein Cyclin D1. Cyclins function as regulators of CDKs (Cyclin-dependent kinases). This cyclin forms a complex with and functions as a regulatory subunit of CDK4 or CDK6, whose activity is required for cell cycle G1/S transition. Cyclin D1 has been shown to interact with Retinoblastoma (Rb), which is a tumor suppressor protein. Cyclin D1 expression should be down regulated in HPV-positive head and neck squamous cell carcinoma as a result of pRb suppression. |
